# The NAD^+^ precursor nicotinamide riboside decreases exercise performance in rats

**DOI:** 10.1186/s12970-016-0143-x

**Published:** 2016-08-02

**Authors:** Ioannis A. Kourtzidis, Andreas T. Stoupas, Ioannis S. Gioris, Aristidis S. Veskoukis, Nikos V. Margaritelis, Maria Tsantarliotou, Ioannis Taitzoglou, Ioannis S. Vrabas, Vassilis Paschalis, Antonios Kyparos, Michalis G. Nikolaidis

**Affiliations:** 1 Department of Physical Education and Sport Science at Serres, Aristotle University of Thessaloniki, Agios Ioannis, Serres, 62110 Greece; 2 Intensive Care Unit, 424 General Military Hospital of Thessaloniki, Thessaloniki, Greece; 3 School of Health Sciences, Faculty of Veterinary Medicine, Aristotle University, Thessaloniki, Greece; 4 Department of Physical Education and Sport Science, University of Thessaly, Trikala, Greece; 5 Department of Health Sciences, School of Sciences, European University Cyprus, Nicosia, Cyprus

**Keywords:** NAD^+^, NADP^+^, Nicotinamide riboside, Exercise, Performance

## Abstract

**Background:**

Nicotinamide adenine dinucleotide (NAD^+^) and its phosphorylated form (NADP^+^) are key molecules in ubiquitous bioenergetic and cellular signaling pathways, regulating cellular metabolism and homeostasis. Thus, supplementation with NAD^+^ and NADP^+^ precursors emerged as a promising strategy to gain many and multifaceted health benefits. In this proof-of-concept study, we sought to investigate whether chronic nicotinamide riboside administration (an NAD^+^ precursor) affects exercise performance.

**Methods:**

Eighteen Wistar rats were equally divided in two groups that received either saline vehicle or nicotinamide riboside at a dose of 300 mg/kg body weight/day for 21 days via gavage. At the end of the 21-day administration protocol, both groups performed an incremental swimming performance test.

**Results:**

The nicotinamide riboside group showed a tendency towards worse physical performance by 35 % compared to the control group at the final 10 % load (94 ± 53 s for the nicotinamide riboside group and 145 ± 59 s for the control group; *P* = 0.071).

**Conclusion:**

Our results do not confirm the previously reported ergogenic effect of nicotinamide riboside. The potentially negative effect of nicotinamide riboside administration on physical performance may be attributed to the pleiotropic metabolic and redox properties of NAD^+^ and NADP^+^.

**Electronic supplementary material:**

The online version of this article (doi:10.1186/s12970-016-0143-x) contains supplementary material, which is available to authorized users.

## Background

There is growing research interest in studying the physiological effects of nicotinamide adenine dinucleotide (NAD^+^) given that increased NAD^+^ levels are related to an increase in longevity [1] and NAD^+^ levels fluctuate in response to exercise and nutritional stimuli [2]. NAD^+^ is continuously consumed as a substrate in adenosine diphosphate (ADP)-ribosylation, cyclization, and deacylation reactions that influence many physiological processes [3]. The phosphorylated and reduced form of NAD^+^ (i.e., NADPH) has also emerged as a crucial molecule in the maintenance of a pool of reducing equivalents, which is essential to counteract oxidative damage and for other detoxifying reactions [4]. These functions highlight the need of NAD^+^ and NADP^+^ pathways in the maintenance of cellular homeostasis.

Based on this evidence, boosting the NAD^+^ and NADP^+^ pools via providing precursor molecules might have multifaceted health benefits and therapeutic applications. Nicotinamide riboside has been much publicized in the media as a novel NAD^+^-booster and an exercise-mimetic supplement. In the only relevant exercise study, nicotinamide riboside was demonstrated to induce a marginally non-significant increase in endurance performance in low-fat diet mice, while this tendency was accentuated and became significant under high-fat diet conditions [5]. In a proof-of-concept study, we sought to investigate whether chronic nicotinamide riboside administration affects exercise performance.

## Methods

### Rats

Eighteen male Wistar rats *(Rattus norvegicus)* (*N* = 18) were used in the study (4 months old). Rats were randomly divided into a control group and a nicotinamide riboside group (9 rats each). The animals were housed under a 12 h light:12 h dark cycle, controlled temperature (21–23 °C) and controlled humidity (50–70 %). Commercial rat chow (#160466; Biozois SA, Greece) and tap water were provided ad libitum. All rats were acclimatized in the animal facility and familiarized to swimming. All procedures were in accordance with the European Union guidelines for the care and use of laboratory animals, as well as the “Principles of laboratory animal care” (NIH publication No. 86–23, revised 1985). The project was reviewed and approved by the institutional review board and the appropriate state authority.

### Nicotinamide riboside administration

Nicotinamide riboside (NAD^+^ Cell Regenerator™, Life Extension®, Fort Lauderdale, US) was administered daily for 21 days via gavage at a dose of 300 mg/kg body weight. The dose was chosen based on the study by Conze et al. [6], who reported that 300 mg/kg body weight of nicotinamide riboside did not cause any adverse events. The rats in the control group received a saline vehicle. Individual doses were freshly prepared daily for each rat separately just before the gavage procedure.

### Incremental swimming performance test

The rats were removed from the vivarium to the swimming tanks immediately prior to the performance test after an overnight fast. Rats swam individually in water tanks. The performance test took place between 09:00 and 11:00. Rats swam individually until exhaustion at a water temperature of 34 °C. An incremental load was adjusted at the base of their tail. In particular, a load equal to 2 % of the rats’ body weight was adjusted for the first 4 min and then loads equal to 3.5 % and 5 % of the rats’ body weight were adjusted for the next 8 min (4 min each). A final load equal to 10 % of the rats’ body weight was used and rats were left to swim until exhaustion. A rat was considered to have reached exhaustion when it exhibited loss of coordinated movements and failure to return to the surface within 10 s three consecutive times.

### Statistical analysis

A *t*-test for independent samples was performed to compare the time-to-exhaustion performance between the two groups (mean ± SD). To determine the meaningfulness of the difference in exercise performance between the groups, the effect size was calculated as the difference in time trial between the two groups divided by the mean standard deviation of the two groups. According to a modified Cohen scale (http://www.sportsci.org), effect sizes of 0.2, 0.6, 1.2, 2.0, and 4.0 were considered small, moderate, large, very large, and nearly perfect, respectively. For comparison, the values of the original Cohen scale are 0.2 for small, 0.5 for moderate, and 0.8 for large effects.

## Results

**Fig. 1:**
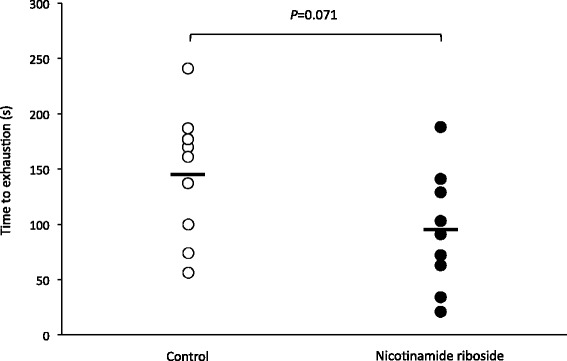
Time to exhaustion in the incremental swimming performance test. Open circles indicate the control group and closed circles indicate the nicotinamide riboside group. The horizontal lines indicate the mean value of each group

The nicotinamide riboside group showed a tendency towards worse physical performance by 35 % compared to the control group at the final 10 % load (94 ± 53 s for the nicotinamide riboside group and 145 ± 59 s for the control group; *P* = 0.071) (Fig. 1, Additional file 1). The effect size calculated was 0.91, which is considered from moderate to large.

## Discussion

NAD^+^ and NADP^+^ are key molecules in ubiquitous bioenergetic and cellular signaling pathways, regulating thereby cellular metabolism and homeostasis. This fueled the interest of many researchers to investigate whether increasing the levels of NAD^+^ and NADP^+^ can enhance exercise performance. Many NAD^+^ and NADP^+^ precursors have developed; yet, all were accompanied by side effects such as severe flushing [7]. A recently identified NAD^+^ precursor, namely nicotinamide riboside, has been demonstrated to increase the levels of NAD^+^ in the absence of any adverse effects [7]. With regard to exercise performance, only one study has been published utilizing nicotinamide riboside and reported marginally non-significant ergogenic effects in low-fat diet mice (approximately 10 % increase in distance run, *P* = 0.08), while this effect became significant in their high-fat diet counterparts (approximately 36 % increase in distance run, *P* < 0.05) [5]. Contrary to this evidence, our findings indicate that administration with nicotinamide riboside decreased (though marginally non-statistically significant) exercise performance in rats. The exact sources of this disagreement are unclear, however, methodological differences such as the animal model (i.e., rats vs. mice), type of exercise (i.e., swimming vs. treadmill running) and route of nicotinamide riboside administration (i.e., gavage vs. food mixture) may have potentially accounted for the conflicting results. On the other hand, our results are in line with some of the studies investigating the effect of another NAD^+^ precursor, namely nicotinic acid, on exercise performance [8, 9]. In particular, the authors of those studies demonstrated that nicotinic acid impaired the ability for prolonged exercise. Despite the fact that nicotinamide riboside is currently preferred compared to nicotinic acid (due to the absence of side effects), both precursors almost similarly increase NAD^+^ levels in most cell types and tissues [5], while they likely share common pathways for NAD^+^ synthesis [7].

Based on the similar effects of nicotinic acid and nicotinamide riboside on NAD^+^ metabolism, the impairments in exercise performance observed in our study may stem from the same sources as in the studies used nicotinic acid [8, 9]. These studies found that nicotinic acid reduced exercise-induced increases in plasma free fatty acids. Therefore, it is likely that nicotinamide riboside decreased fatty acid oxidation during exercise leading to an earlier fatigue. In addition, the redox properties of NAD^+^ and NADP^+^ could also provide a plausible explanation for the impaired performance observed, namely by disrupting redox homeostasis [10]. In particular, nicotinamide riboside administration may have altered redox homeostasis leading cells to a more reductive (non-optimal) state, according to the hormetic theory of reactive oxygen and nitrogen species activity [11]. This is in line with other recent studies stressing the potential detrimental effects of redox-related supplements on exercise capacity [12]. Evidently, these hypotheses are speculative and are currently being investigated in order to reveal the potential metabolic and redox mechanisms involved in our finding. Further studies are warranted to acquire mechanistic insights on the effect of nicotinamide riboside on exercise performance.

## Conclusion

Chronic administration of the NAD^+^ precursor nicotinamide riboside tended to decrease physical performance in rats. We believe that this finding is important and timely and adds to the expanding literature showing that altering metabolic and redox homeostasis via exogenously administered agents may lead to adverse and not necessarily beneficial or neutral effects.

## Additional file

Additional file 1: Raw data of time to exhaustion in the incremental swimming test for the control and the nicotinamide riboside group. (XLSX 9 kb)

## Abbreviation

NAD^+^: Nicotinamide adenine dinucleotide
